# The Week's Work

**Published:** 1910-08-27

**Authors:** 


### THE WEEK'S WORK.

### HOSPITAL AND CRIME.

"The Women's Hospital at Melbourne (writes an Australian correspondent) is frequently asked by the police for the names and addresses of the mothers of illegitimate children born within the institution during the month or fortnight preceding the query. This happens when an infant has been found murdered or deserted. The women of the Committee held that the hospital is a sanctuary for these mothers, and have often refused to give such information. At present the police are particularly bitter against the Committee. In view of this continual clashing of forces it would be interesting to know, through The Hospital, what is the usage in England." We have always understood that hospital authorities in this country invariably refuse to supply any such information, just as private practitioners refuse. Neither the Committee nor a general practitioner has the legal right to give such information, and it seems to us that the Committee's action will have the cordial approval of all hospital workers. Hospitals have no justification for refusing admittance to such lying-in patients as our correspondent refers to, and certainly no justification whatever for treating them differently from other patients in so far as infringing the professional etiquette which forbids the medical attendant from divulging secrets learned in the course of his attendance is concerned.

### THE BRITISH INSTITUTE OF SOCIAL SERVICE.

This very useful association is working so quietly and unostentatiously that its last annual report, showing what has been accomplished during the past twelve months, contains much that comes as a surprise to the reader who has not been specially interested in social work. It is a pity that the association is so little known to hospital workers. We are of opinion that its activities would be helped and its scope of usefulness extended if it came into closer touch with the hospitals than it does at present. The Institute, which is entirely relying upon voluntary support, and does not, like its well-known prototype at Milan, receive State aid, has answered many queries and compiled working bibliographies on a number of interesting social questions, chiefly relating to child labour, education, and relief. We excerpt the following from the report, as it shows a phase of the society's activities which is of special interest to institutional workers: "The Joint Committee on the Abolition of Half-time Labour was formed during the year, its objects being—(1) To carry into law the proposals of the Inter-Departmental Committee on Partial Exemption from School Attendance, and (2) to assist the Half-time Council in its efforts to abolish all exemptions from school attendance up to the age of fourteen. A considerable amount of information has been collected and prepared on various careers for paid social service and of opportunities for volunteers in social work for Mr. Malcolm Spencer, who embodied the facts and help thus given him in 'Social Reclamation.' Mr. Spencer in this book bears testimony to the services rendered him by the Institute. Help was given to Professor Macgregor, of Leeds University, in the arrangement of a course of lectures on the 'Social Relations of Industry,' 'City Problems,' 'Country Problems,' 'Wages and Employment,' 'Wages in Organised Trades,' 'Wages in Unorganised Trades,' and 'Unemployment.' At the request of Mr. M. Szanto, the Director of the Musée Social in Budapest, the Institute made a collection of between seven hundred and eight hundred reports and leaflets, as well as photographs and slides, on ameliorative work amongst children in England for a permanent exhibition in Budapest."

### THE OBJECTS OF THE B.I.S.S.

The Institute exists, as its leaflets concisely put it, to collect and register information relating to all forms of social service and to put it within the reach of all concerned in the improvement and elevation of our national life. In these ways it hopes to assist those engaged in initiating social effort to do it on the most approved lines and to prevent overlapping with existing organisations. Hospital workers will find that they, at some time or other, need information on special points, and they would do well to bear the Institute in mind and to apply to the Secretary when they wish any information in connection with social service work. But even better than that, we should suggest, is to become a member of the B.I.S.S. The membership fee is only five shillings per annum (which entitles the member to a free copy of the journal, "Progress"), and the Institute wants support both in money and in men and women who will interest themselves in its aims.

### THE CHESTER INFIRMARY.

The Pageant which was recently held at Chester has been successful not only from an artistic and spectacular point (as every pageant ought to be), but equally so from a financial point of view. There is, we understand, a substantial surplus, and the Pageant Committee has now the pleasant but apparently somewhat difficult task of spending this surplus to the best advantage. The Mayor, Alderman D. L. Hewitt, has pressed the claims of the child workers who helped to ensure the success of the show by their unstinted hard work, and has proposed that part of the profits should be devoted to giving these children a trip and a tea. This has been done, but there is still a sum of money remaining in the Committee's hands. Alderman Hewitt now comes forward, in a letter to the local Press, with the suggestion that the Committee should consider the advisability of voting the surplus as a nucleus for the alteration, rebuilding, or erection of a new hospital. "If the subscribers," he remarks in his letter, "to the

Pageant would allow their subscriptions to be also devoted to this noble work, a sum could be raised worthy of the city and county, and might form a memorial to the ever-revered King Edward VII., whose life-long interest in hospitals would be fittingly commemorated." With that suggestion we cordially agree. The old infirmary at Chester is in many ways unworthy of the town, which requires additional hospital accommodation as well. We trust the Pageant Committee will seriously consider the advisability of adopting the Mayor's suggestion, and that its decision will have the unanimous support of the subscribers as well.

### THE DRUG MARKET.

Business in the drug market continues quiet, and few changes in price of special importance have taken place. Rochelle salts and seidlitz powder are dearer. Cream of tartar and tartaric acid still show a slightly advancing tendency. Hydrastis is rather dearer. Castor oil tends somewhat cheaper. Cod-liver oil is, if anything, slightly dearer, but no material advance in price is expected. Peppermint oil is fetching rather higher prices, but business is restricted to small orders. Opium continues to tend lower, and morphine is obtainable at lower rates. Codeine is unchanged. Senega tends cheaper.

